# Ultrafast Dynamics Across Pressure‐Induced Electronic State Transitions, Fluorescence Quenching, and Bandgap Evolution in CsPbBr_3_ Quantum Dots

**DOI:** 10.1002/advs.202308016

**Published:** 2024-02-02

**Authors:** Lin Chen, Ya Chu, Xiaxia Qin, Zhijian Gao, Guozhao Zhang, Haiwa Zhang, Qinglin Wang, Qian Li, Haizhong Guo, Yinwei Li, Cailong Liu

**Affiliations:** ^1^ School of Physics Science & Information Technology Liaocheng University Liaocheng 252059 P. R. China; ^2^ Key Laboratory of Material Physics Ministry of Education School of Physics and Microelectronics Zhengzhou University Zhengzhou 450052 P. R. China; ^3^ Laboratory of Quantum Functional Materials Design and Application School of Physics and Electronic Engineering Jiangsu Normal University Xuzhou 221116 P. R. China

**Keywords:** bandgap, electron state, fluorescence, pressure, ultrafast dynamic

## Abstract

This work investigates the impact of pressure on the structural, optical properties, and electronic structure of CsPbBr_3_ quantum dots (QDs) using steady‐state photoluminescence, steady‐state absorption, and femtosecond transient absorption spectroscopy, reaching a maximum pressure of 3.38 GPa. The experimental results indicate that CsPbBr_3_ QDs undergo electronic state (ES) transitions from ES‐I to ES‐II and ES‐II to ES‐III at 0.38 and 1.08 GPa, respectively. Intriguingly, a mixed state of ES‐II and ES‐III is observed within the pressure range of 1.08–1.68 GPa. The pressure‐induced fluorescence quenching in ES‐II is attributed to enhanced defect trapping and reduced radiative recombination. Above 1.68 GPa, fluorescence vanishes entirely, attributed to the complete phase transformation from ES‐II to ES‐III in which radiative recombination becomes non‐existent. Notably, owing to stronger quantum confinement effects, CsPbBr_3_ QDs exhibit an impressive bandgap tuning range of 0.497 eV from 0 to 2.08 GPa, outperforming nanocrystals by 1.4 times and bulk counterparts by 11.3 times. Furthermore, this work analyzes various carrier dynamics processes in the pressure‐induced bandgap evolution and electron state transitions, and systematically studies the microphysical mechanisms of optical properties in CsPbBr_3_ QDs under pressure, offering insights for optimizing optical properties and designing novel materials.

## Graphical abstract

CsPbBr_3_ quantum dots (QDs) undergo electronic state (ES) transitions from ES‐I to ES‐II to ES‐III under high pressure, accompanied by changes in fluorescence and bandgap. In the pressure range of 1.08–1.68 GPa, ES‐II and ES‐III coexist in the QDs. Pressure‐induced fluorescence quenching and disappearance are behaviors of ES‐II. Above 1.68 GPa, ES‐III is no radiative recombination.

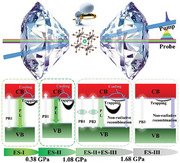

## 1 Introduction

CsPbBr_3_ is a typical perovskite solar cell material that possesses remarkable characteristics,^[^ ^1^ ^]^ including excellent broadband absorption,^[^ ^1^, ^2^ ^]^ tunable fluorescence wavelengths,^[^ ^2^, ^3^ ^]^ high fluorescence quantum yield,^[^ ^2^, ^4^, ^5^ ^]^ efficient charge transfer,^[^ ^6^ ^]^ narrow full width at half maximum, and high color purity of emission.^[^ ^7^, ^8^, ^9^, ^10^ ^]^ It has attracted significant attention for its potential application in fields such as solar cells,^[^ ^1^, ^11^, ^12^ ^]^ light‐emitting diodes,^[^ ^13^, ^14^, ^15^ ^]^ and photodetectors.^[^ ^16^, ^17^ ^]^ Due to the presence of the quantum confinement effects, CsPbBr_3_ quantum dots (QDs) and nanocrystals (NCs) exhibit superior optical and optoelectronic properties compared to bulk counterparts.^[^ ^18^, ^19^, ^20^, ^21^, ^22^, ^23^ ^]^ Improving the optical performance of CsPbBr_3_ nanomaterials has been a research hotspot.

The regulation of size and pressure is an effective method to optimize the material structures and enhance performance.^[^ ^7^, ^24^, ^25^, ^26^, ^27^, ^28^, ^29^, ^30^, ^31^, ^32^ ^]^ In recent years, researchers have conducted extensive studies on the crystal structure and optical properties of the CsPbBr_3_ bulk counterparts and CsPbBr_3_ NCs under high pressure. Zhang et al. discovered an isostructural phase transition (orthorhombic phase) in CsPbBr_3_ bulk counterparts near 1.0 GPa, which was accompanied by an anomalous discontinuous evolution of the bandgap, with a tunable range of 0.044 eV.^[^ ^33^ ^]^ In addition, the fluorescence gradually quenched under pressure and disappeared at 2.5 GPa. Xiao et al. also observed an isostructural phase transition (orthorhombic phase) in CsPbBr_3_ NCs near 1.19 GPa, as well as significant pressure‐modulated bandgap and pressure‐induced fluorescence quenching (the fluorescence disappeared at 1.36 GPa).^[^ ^34^ ^]^ Furthermore, Gao et al. revealed electronic state (ES) transitions in CsPbBr_3_ NCs (average particle size of 10.4 nm) from ES‐I to ES‐II at 0.38 GPa and from ES‐II to ES‐III at 1.08 GPa, accompanied by anomalous discontinuous evolutions of the bandgap and the carrier lifetime.^[^ ^35^ ^]^ They also observed the pressure‐induced fluorescence quenching, with the fluorescence disappearing at 1.38 GPa. By comparison, the electronic state transition from ES‐II to ES‐III at 1.08 GPa corresponds to the isostructural phase transition mentioned before, while the transition from ES‐I to ES‐II at 0.38 GPa has not been discovered in CsPbBr_3_ bulk counterparts. Our research team investigated the ultrafast carrier dynamics of the CsPbBr_3_ bulk counterparts through in situ high‐pressure femtosecond transient absorption (fs‐TA) spectroscopy.^[^ ^36^ ^]^ We discovered the electronic state transition from ES‐II to ES‐III but not the transition from ES‐I to ES‐II. Moreover, pressure effectively reduced the bandgap and prolonged the hot carrier relaxation and Auger recombination before the phase transition.^[^ ^36^ ^]^ Based on the above observations, it can be concluded that CsPbBr_3_ NCs exhibit different pressure‐induced phase transition pathways, a larger tunable range of bandgap, and a lower pressure for fluorescence disappearing compared to CsPbBr_3_ bulk counterparts. CsPbBr_3_ QDs and NCs are similar in size, so they both have strong quantum confinement effects, high surface energy, and excellent chemical stability.^[^ ^25^ ^]^ Therefore, CsPbBr_3_ QDs and NCs have superior optical properties compared to their bulk counterparts. However, there hasn't been a systematic investigation on the ultrafast dynamics of the pressure‐induced electronic state transitions, fluorescence quenching, and bandgap evolution in CsPbBr_3_ QDs and NCs.

In situ high‐pressure X‐ray diffraction (XRD), steady‐state photoluminescence (PL), and steady‐state absorption (Abs) spectroscopy based on Diamond Anvil Cell (DAC) are powerful tools for studying the structural phase transitions, the variation in fluorescence intensity, and the evolution of bandgap under pressure.^[^ ^37^, ^38^, ^39^, ^40^, ^41^, ^42^, ^43^, ^44^, ^45^, ^46^, ^47^, ^48^, ^49^, ^50^, ^51^, ^52^, ^53^, ^54^ ^]^ However, these techniques have limitations in characterizing certain complex physical processes such as the pressure‐induced electronic state transitions,^[^ ^35^, ^55^ ^]^ charge transfer,^[^ ^56^, ^57^ ^]^ and proton transfer.^[^ ^58^, ^59^ ^]^ Additionally, they are unable to provide insights into the physical mechanisms behind the phenomena such as the pressure‐induced fluorescence quenching,^[^ ^60^, ^61^ ^]^ enhancement,^[^ ^62^, ^63^ ^]^ and aggregation‐induced emission.^[^ ^64^, ^65^ ^]^ In recent years, with the advancement of fs‐TA spectroscopy detection techniques, they have also been introduced into the field of high‐pressure science to investigate the ultrafast dynamics under pressure, including the excited state charge carriers transitions,^[^ ^66^ ^]^ relaxation,^[^ ^67^, ^68^, ^69^, ^70^ ^]^ recombination,^[^ ^71^, ^72^ ^]^ electron transfer,^[^ ^73^, ^74^ ^]^ and fluorescence resonance energy transfer.^[^ ^75^, ^76^ ^]^ By combining in situ high‐pressure XRD, PL, Abs, and fs‐TA spectroscopy detection techniques, the intricate connection between the microscopic ultrafast carrier dynamics and macroscopic optical behaviors can be explored comprehensively.^[^ ^77^ ^]^ This advancement enabled the systematic study of optical physics mechanisms in materials.

In this study, in situ high‐pressure steady‐state PL, Abs, and fs‐TA spectroscopy experiments were carried out to explore the PL intensity, bandgap, and ultrafast dynamics of CsPbBr_3_ QDs with an average particle size of 7.6 nm under high pressure. By analyzing the experimental results and comparing them with those of CsPbBr_3_ NCs with an average particle size of 10.4 nm and CsPbBr_3_ bulk counterparts, the connection between the microscale ultrafast dynamics of excited‐state carriers and the macroscopic optical behavior was established, which revealed the physical mechanisms of the electronic state transitions, fluorescence quenching, and bandgap evolution in CsPbBr_3_ QDs under pressure.

## 2 Results and Discussion

### 2.1 Characterization of CsPbBr_3_ QDs at Ambient Pressure

The CsPbBr_3_ QDs sample displayed a uniform morphology in the high‐resolution transmission electron microscopy image, as shown in Figure S1a (Supporting Information). The corresponding size distribution was obtained via Gaussian fitting, revealing an average size of 7.6±0.1 nm, as depicted in Figure S1b (Supporting Information). In Figures S1c,d (Supporting Information), the fluorescence peak of CsPbBr_3_ QDs located at ≈ 510 nm and the first excitonic absorption edge appeared at ≈ 498 nm under ambient conditions.^[^ ^78^ ^]^ The bandgap value was calculated to be 2.40 eV using the Tauc plot extrapolation,^[^ ^79^ ^]^ which was consistent with the results reported by Protesescu et al..^[^ ^24^ ^]^

### 2.2 Pressure‐Induced Fluorescence Quenching of CsPbBr_3_ QDs

**Figure 1:**
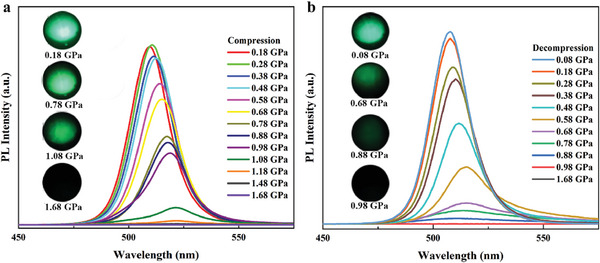
In situ high‐pressure steady‐state PL spectra of CsPbBr_3_ QDs. a) Steady‐state PL spectra during compression, with insets showing the micrographs of CsPbBr_3_ QDs in the sample chamber at 0.18, 0.78, 1.08, and 1.68 GPa. b) Steady‐state PL spectra during decompression, with insets showing the micrographs of CsPbBr_3_ QDs in the sample chamber at 0.98, 0.88, 0.68, and 0.08 GPa.

Pressure‐induced fluorescence quenching refers to the phenomenon where the fluorescence intensity of a material decreases or disappears under high pressure conditions.^[^ ^60^, ^80^ ^]^ To study the effect of pressure on the fluorescence radiative ability of CsPbBr_3_ QDs, we carried out in situ high‐pressure steady‐state PL spectroscopy measurements on the CsPbBr_3_ QDs. **Figure** **1**a shows steady‐state PL spectra of the CsPbBr_3_ QDs during compression. At 0.18 GPa, CsPbBr_3_ QDs exhibit green emission, and the fluorescence intensity progressively diminishes until it vanishes as the pressure increases. From 0.18 to 0.28 GPa, the fluorescence peak undergoes a slight redshift, and the fluorescence intensity is slightly enhanced. From 0.38 to 1.08 GPa, the fluorescence peak of CsPbBr_3_ QDs continues to redshift, but the fluorescence intensity begins to diminish gradually, which may be attributed to the electron state transition from ES‐I to ES‐II.^[^ ^35^ ^]^ This indicates a gradual reduction in the fluorescence radiative ability of the CsPbBr_3_ QDs under pressure, accompanied by a decrease in the recombination of the participating carriers, which is consistent with the overall trend observed in the bulk counterparts and NCs.^[^ ^33^, ^34^, ^35^ ^]^ Above 1.08 GPa, the fluorescence intensity decreases significantly with the increase of pressure, and the fluorescence disappears at 1.68 GPa. This is related to the electron state transition that occurs from ES‐II to ES‐III.^[^ ^35^ ^]^ In Figure 1b, during decompression, the emission color gradually recovers to green. When the pressure decreases to 0.88 GPa, the fluorescence peaks start to appear. From 0.88 to 0.08 GPa, a blueshift in the fluorescence peak is observed, and the intensity continues to enhance. When the pressure reaches 0.08 GPa, the fluorescence peak is located around 509 nm, which reveals that the changes in PL properties of CsPbBr_3_ QDs under pressure are reversible. However, there is a pressure relaxation of 0.8 GPa between the disappearance of the pressure‐induced fluorescence peak and the reappearance of the pressure‐relieved fluorescence peak.

By comparison, it can be seen that the overall trend of the fluorescence behavior under high pressure of CsPbBr_3_ QDs, NCs,^[^ ^35^ ^]^ and bulk counterparts^[^ ^33^ ^]^ is consistent. However, due to the quantum confinement effect in QDs, two different phenomena are observed. First, the fluorescence intensity of QDs shows a slight enhancement at 0.28 GPa, while this is not observed in NCs and bulk counterparts. The slight enhancement of pressure‐induced fluorescence intensity is attributed to the fact that QDs have a strong quantum confinement effect, and the enhanced passivation of surface states and defects in smaller QDs under high pressure,^[^ ^62^, ^63^, ^81^ ^]^ increasing in the participating radiative recombination carriers. Second, the fluorescence disappearance is more sensitive to pressure in QDs and NCs compared to the bulk counterparts. As shown in **Table** **1**, the pressure at which fluorescence vanishes for bulk counterparts is 2.5 GPa, while that for NCs, and QDs is 1.38 and 1.68 GPa respectively. Previous studies attributed the pressure‐induced fluorescence disappearance in the CsPbBr_3_ bulk counterparts and NCs to the increase in non‐radiative recombination caused by the ES‐III pressure‐induced amorphization,^[^ ^33^, ^34^, ^35^, ^82^ ^]^ but require further investigation. Therefore, the physical mechanism of the pressure‐induced fluorescence quenching of the CsPbBr_3_ bulk counterparts, NCs, and QDs is still unclear.

**Table 1:** The fluorescence disappearance pressure of CsPbBr_3_ bulk counterparts, NCs, and QDs.

Materials	Fluorescence disappearance pressure
CsPbBr_3_ bulk counterparts^[^ ^33^ ^]^	2.50 GPa
CsPbBr_3_ NCs (10.4±1.8 nm)^[^ ^35^ ^]^	1.38 GPa
CsPbBr_3_ QDs (7.6±0.1 nm)	1.68 GPa

### 2.3 Pressure‐Induced Bandgap Evolution of CsPbBr_3_ QDs

**Figure 2:**
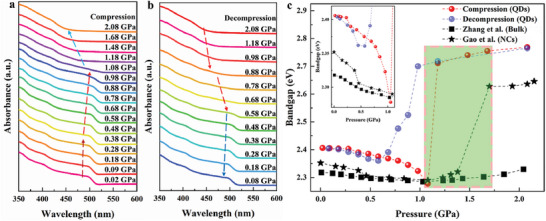
In situ high‐pressure steady‐state Abs spectra of CsPbBr_3_ QDs. a) Steady‐state Abs spectra during compression. b) Steady‐state Abs spectra during decompression. c) Pressure‐dependent bandgaps of CsPbBr_3_ QDs during compression (red ball) and decompression (red ball), along with the pressure‐dependent bandgaps of CsPbBr_3_ bulk counterparts (black square) and CsPbBr_3_ NCs (black pentagram) during compression.^[^ ^33^, ^35^ ^]^ The inset illustrates the pressure‐dependent bandgap from 0 to 1.1 GPa, the green‐shaded region corresponds to the electron state transition interval from ES‐II to ES‐III in CsPbBr_3_ QDs.

To investigate the changes of the electronic band structure during the pressure‐induced electron state transition in CsPbBr_3_ QDs and further reveal the physical mechanism of the pressure‐induced fluorescence quenching, in situ high‐pressure steady‐state Abs spectroscopy measurements on CsPbBr_3_ QDs were conducted. As shown in **Figure** **2**a, there are two distinct discontinuous changes in the absorption edge of CsPbBr_3_ QDs within the pressure range of 0.02 to 2.08 GPa. At ≈ 0.38 GPa, the absorption edge of CsPbBr_3_ QDs starts to have a significant redshift as the pressure increases. Then, above 1.08 GPa, the absorption edge undergoes a continuous blueshift with increasing pressure. These changes correspond to the electronic state transitions of CsPbBr_3_ QDs from ES‐I to ES‐II and from ES‐II to ES‐III respectively,^[^ ^35^ ^]^ which are also consistent with our steady‐state PL measurements. Additionally, above 1.08 GPa, the first excitonic absorption edge starts to weaken, and eventually disappears completely at 1.68 GPa. This agrees well with the substantial weakening and disappearance of fluorescence observed in Figure 1a. Figure 2b displays steady‐state Abs spectra of CsPbBr_3_ QDs during the decompression process. When the pressure is released from 2.08 to 0.88 GPa, the first exciton absorption edge reappears,^[^ ^78^ ^]^ which corresponds to the re‐emergence of the fluorescence peak at 0.88 GPa shown in Figure 1b. From 0.88 to 0.58 GPa, the absorption edge undergoes a redshift, indicating the electron state transition from ES‐III back to ES‐II. Finally, as the pressure is further decreased from 0.58 to 0.08 GPa, the absorption edge undergoes blueshift, returning to the initial ES‐I. This confirms the reversibility of the electron state transition of CsPbBr_3_ QDs under high pressure.

The bandgap of CsPbBr_3_ QDs at different pressures was calculated by the Tauc plot extrapolation method.^[^ ^79^, ^83^ ^]^ As shown in Figure 2c, from 0.02 to 1.08 GPa, the bandgap value of CsPbBr_3_ QDs decreases with increasing pressure. This is attributed to the shortening of Pb‐Br bond lengths and the enhanced coupling between the Pb 6s and Br 4p orbitals under pressure, resulting in the narrowing of the bandgap between the valence band maximum (VBM) and the conduction band minimum (CBM).^[^ ^33^, ^34^, ^35^, ^36^, ^84^, ^85^ ^]^ The abrupt decrease of the bandgap value at 0.38 GPa (as shown in the inset of Figure 2c) corresponds to the electronic state transition from ES‐I to ES‐II.^[^ ^35^ ^]^ From 1.08 to 2.08 GPa, the bandgap value increases with increasing pressure, which is due to the distortion of octahedral [PbBr_6_]4^−^ under high pressure, leading to a reduction in Pb‐Br‐Pb bond angles and a weakening of coupling strength between Pb 6s and Br 4p orbitals, resulting in the widening of the bandgap between VBM and CBM.^[^ ^33^, ^34^, ^35^, ^36^, ^84^, ^85^ ^]^ The significant increase in the bandgap value at 1.18 GPa corresponds to the electronic state transition from ES‐II to ES‐III.^[^ ^35^ ^]^ During decompression, the changes in bandgap with pressure are reversible.

Through comparison, the bandgaps of CsPbBr_3_ bulk counterparts, NCs, and QDs have similar evolution under pressure, and they all undergo a structural phase transition around 1.08 GPa. However, pressure regulates many of their physical properties differently due to different sizes. First, from 0 to 2.08 GPa, QDs have a bandgap tuning range of 0.497 eV, which is 1.4 times that of NCs and 11.3 times that of bulk counterparts, as shown in **Table** **2**. This is because the smaller the size of CsPbBr_3_, the stronger the quantum confinement effect,^[^ ^24^, ^86^ ^]^ and the more discrete the energy level structure,^[^ ^87^, ^88^ ^]^ which leads to more significant changes in the bandgap under pressure. Second, due to the strong quantum confinement effects, QDs and NCs exhibit discontinuous changes in the bandgap at 0.38 GPa, indicating an electron state transition from ES‐I to ES‐II, which is absent in the bulk counterparts.^[^ ^24^ ^]^ Nevertheless, the electronic state transition interval from ES‐II to ES‐III exists in the CsPbBr_3_ QDs, NCs, and bulk counterparts, and ES‐III is considered to be radiate‐free fluorescence. The disappearance of the pressure‐induced fluorescence of the CsPbBr_3_ bulk counterparts and NCs in previous studies is attributed to the increase of non‐radiative recombination caused by the pressure‐induced amorphism in ES‐III.^[^ ^33^, ^34^, ^35^, ^82^ ^]^

**Table 2:** The bandgap tuning range and octahedral distortion pressure of CsPbBr_3_ bulk counterparts, NCs, and QDs under high pressure.

Materials	Octahedral distortion pressure	Bandgap tuning range
CsPbBr_3_ bulk counterparts^[^ ^33^ ^]^	1.0 GPa	0.044 eV
CsPbBr_3_ NCs (10.4±1.8 nm)^[^ ^35^ ^]^	1.08 GPa	0.355 eV
CsPbBr_3_ QDs (7.6±0.1 nm)	1.08 GPa	0.497 eV

Based on the experimental results of in situ high‐pressure steady‐state Abs spectra,^[^ ^33^, ^34^, ^35^ ^]^ the comparative analysis shows that after the disappearance of the pressure‐induced fluorescence, obvious steady‐state Abs spectra of CsPbBr_3_ QDs, NCs, and bulk counterparts can be still obtained. It is confirmed that CsPbBr_3_ QDs, NCs, and bulk counterparts still maintain a good lattice structure (orthogonal phase), and are not amorphous. These results indicate that the disappearance of the pressure‐induced fluorescence of CsPbBr_3_ QDs, NCs, and bulk counterparts is caused by the complete transformation from ES‐II to non‐fluorescent ES‐III under pressure, rather than the pressure‐induced amorphous in ES‐III. The ES‐III of CsPbBr_3_ NCs at 1.08‐1.38 GPa reported by Gao et al. should be a mixed state of ES‐II and ES‐III.^[^ ^35^ ^]^ This corresponds to the mixed state of CsPbBr_3_ NCs in the 1.19‐1.45 GPa reported by Xiao et al.,^[^ ^34^ ^]^ and that of the CsPbBr_3_ bulk counterparts in the 1.21‐2.03 GPa reported by Zhang et al. and Xiao et al..^[^ ^33^, ^34^ ^]^ It is also consistent with the mixed states of ES‐II and ES‐III in the 1.08‐1.68 GPa of CsPbBr_3_ QDs measured in our work (as shown in the green‐shaded area in Figure 2c). At present, the physical mechanism of the pressure‐induced fluorescence quenching of CsPbBr_3_ QDs, NCs, and bulk counterparts is still not elucidated clearly, which prompts us to investigate ultrafast dynamical behaviors of CsPbBr_3_ QDs under pressure by using in situ high‐pressure fs‐TA spectroscopy.

### 2.4 In Situ High‐Pressure Ultrafast Dynamics of CsPbBr_3_ QDs

The pressure‐induced electronic state transitions, fluorescence quenching, and bandgap evolution of materials are closely related to their ultrafast dynamics.^[^ ^36^, ^56^, ^71^, ^89^, ^90^, ^91^ ^]^ To investigate the ultrafast dynamics of the pressure‐induced electronic state transitions, fluorescence quenching, and bandgap evolution in CsPbBr_3_ QDs, and reveal the physical mechanism of the fluorescence enhancement, quenching, and disappearance of different electronic states under high pressure, we conducted in situ high‐pressure fs‐TA spectroscopy measurements on CsPbBr_3_ QDs. The experimental results were compared with our previous data of the in situ high‐pressure ultrafast dynamics of the CsPbBr_3_ bulk counterparts.^[^ ^36^ ^]^

**Figure 3:**
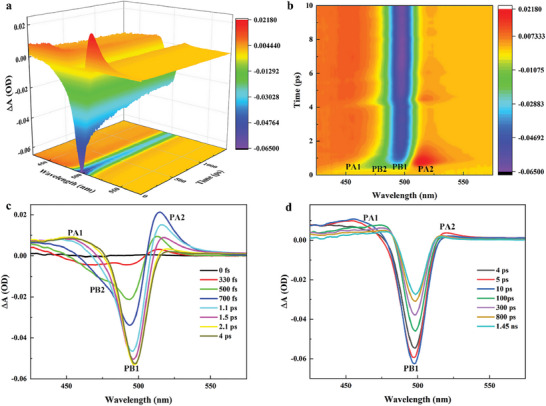
Fs‐TA spectra of CsPbBr_3_ QDs at ambient pressure. a) 3D image plots. b) 2D image plots. c) Time evolution plots in the delay time range of 0 fs‐4 ps. d) Time evolution plots in the delay time range of 4 ps‐1.45 ns.

Fs‐TA spectra of CsPbBr_3_ QDs at ambient pressure (as shown in **Figure** **3**) obtained in our measurement are similar to that of CsPbBr_3_ NCs reported by Anunay Samanta et al..^[^ ^28^ ^]^ Figure 3a shows 3D image plots of fs‐TA spectra, where two positive‐signal peaks are observed near 450 and 520 nm, respectively, and one negative‐signal peak near 500 nm. Figure 3b shows 2D image plots of fs‐TA spectra with a delay time of 0–10 ps. The positive and negative signals are observed in the region of 425 to 575 nm. Figures 3c,d show the evolution of fs‐TA spectra at different delay times (where ΔA is the change in absorption). The broad absorption band located from 425 to 475 nm (positive signal) is labeled PA1. PA1 is attributed to the absorption of the first excited state carrier and is formed by the transition of the lowest excited state carrier to the higher excited state by absorbing pump light energy.^[^ ^28^, ^57^ ^]^ The peaks near 498 nm and acromial peaks near 480 nm (negative signal) are labeled PB1 and PB2, respectively. Among them, PB1 is attributed to the ground‐state bleach and is formed by the weakened absorption of ground‐state carriers to the probe light.^[^ ^28^, ^57^ ^]^ PB2 is attributed to the hot carrier‐induced bleach, and the rapid recovery of PB2 (>700 fs) caused the broadening of the spectral envelope of PA1.^[^ ^95^ ^]^ The broad absorption band in the region of 510 to 575 nm (positive signal) is labeled as PA2, and PA2 is attributed to the absorption of hot carriers, which is formed by the transition of the hot carriers from the absorption of the pump light energy in the lower excited state to the higher excited state.^[^ ^28^, ^57^ ^]^

**Figure 4:**
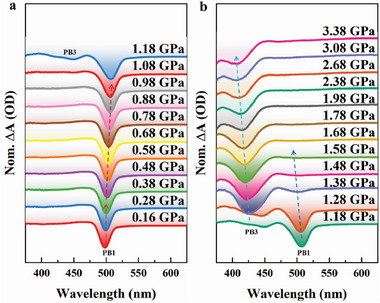
Normalized fs‐TA spectra of CsPbBr_3_ QDs for a 10 ps probe delay under different pressures. a) 0.16–1.18 GPa. b) 1.18–3.38 GPa.

To further study the ultrafast dynamics of excited state carriers in CsPbBr_3_ QDs under pressure, we carried out in situ high‐pressure fs‐TA spectroscopy measurements of CsPbBr_3_ QDs. **Figure** **4** shows normalized fs‐TA spectra of CsPbBr_3_ QDs for a 10 ps probe delay under different pressures. As shown in Figure 4a, from 0.16 to 1.08 GPa, PB1 continuously redshifts with increasing pressure. Above 0.38 GPa, the redshifted trend of PB1 becomes more obvious, which is related to the electronic state transition from ES‐I to ES‐II.^[^ ^35^ ^]^ At 1.18 GPa, the energy value (about 2.3 eV) corresponding to the PB1 signal peak is consistent with the bandgap value of ES‐II, while a new negative‐signal peak appears near 440 nm, and the energy value corresponding to its peak position is ≈ 2.8 eV, which is consistent with the bandgap value of ES‐III in Figure 2c. Therefore, this new negative‐signal peak is regarded as the ground‐state bleach (labeled as PB3) in ES‐III. As shown in Figure 4b, above 1.08 GPa, both PB1 and PB3 continuously blueshift with increasing pressure. The change in PB1 trend and the appearance of PB3 are attributed to the transformation from ES‐II to ES‐III.^[^ ^35^ ^]^ With the increase of pressure, the PB1 signal representing ES‐II gradually weakens, while the PB3 signal representing ES‐III appears and gradually increases. Until 1.68 GPa, the disappearance of PB1 signifies the disappearance of ES‐II, and the mixed state of ES‐II and ES‐III completely transforms into the non‐radiative ES‐III, which is consistent with the disappearance of the fluorescence peak observed in Figure 1a for CsPbBr_3_ QDs.

**Figure 5:**
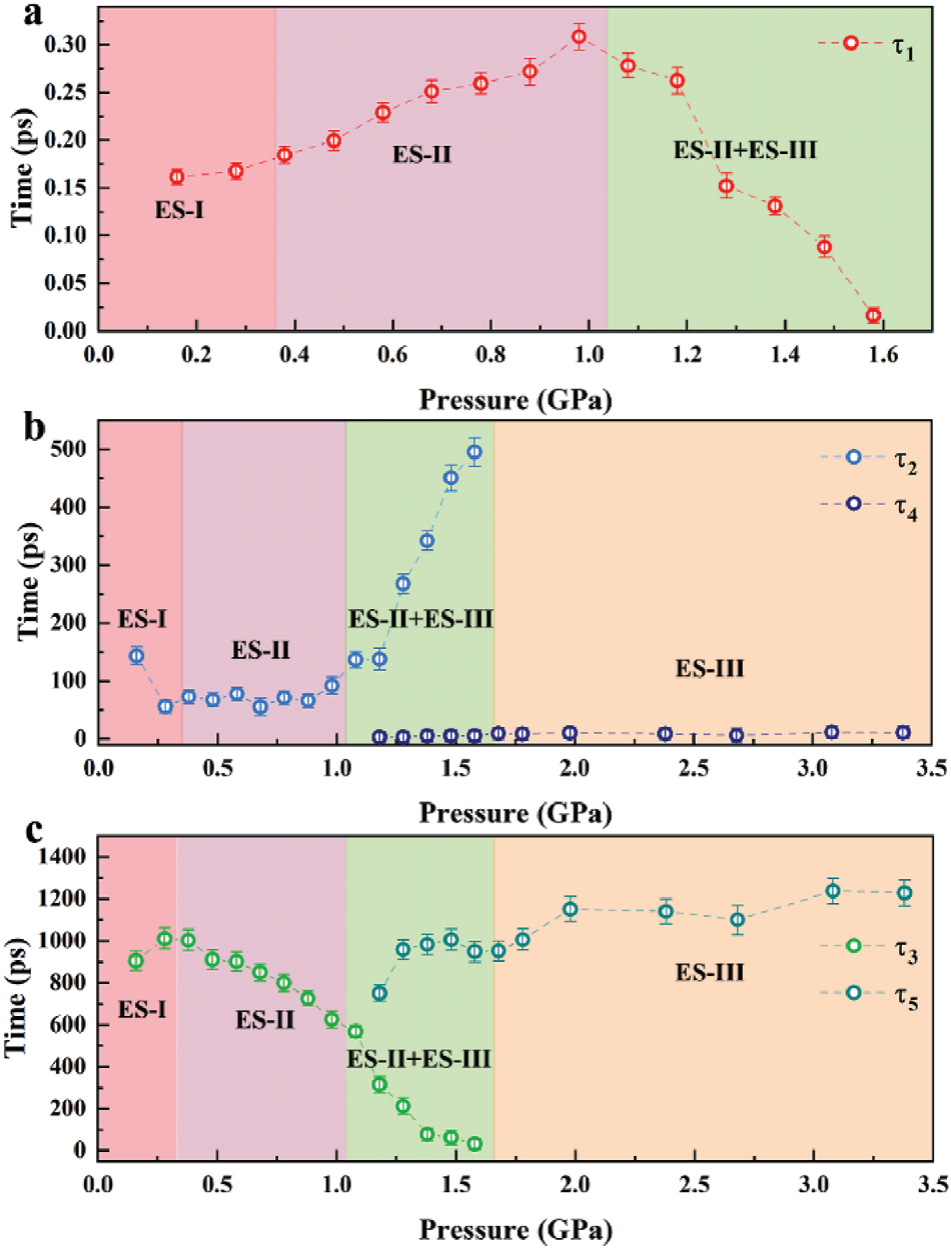
Ultrafast dynamics lifetimes of CsPbBr_3_ QDs carriers at different pressures. a) Hot carrier relaxation lifetimes (τ_1_). b) Carrier lifetimes by defect trapping (τ_2_), carrier lifetimes by surface state trapping (τ_4_). c) Radiative recombination lifetimes (τ_3_), non‐radiative recombination lifetimes (τ_5_).

The global fitting of in situ high‐pressure fs‐TA spectra of CsPbBr_3_ QDs at different pressures was performed for further analysis.^[^ ^92^ ^]^ As shown in **Figure** **5**, the lifetimes for the ultrafast dynamics of the carriers under different pressures were obtained from the fitting results, which include the hot carrier relaxation lifetimes (τ_1_), carrier lifetimes by defect trapping (τ_2_), radiative recombination lifetimes (τ_3_), carrier lifetimes by surface state trapping (τ_4_), and non‐radiative recombination lifetimes (τ_5_).^[^ ^28^, ^93^, ^94^, ^95^ ^]^

From Figure 5a, τ_1_ increases with the increase of pressure from 0.16 to 0.98 GPa. One of the reasons is that the Pb‐Br bond lengths of ES‐I and ES‐II shrink under pressure, and the resulting repulsive force causes the lattice hardening,^[^ ^96^ ^]^ which makes it difficult for the hot carriers to cool down by interacting with the lattice. Another reason is that the decreasing bandgap value (as shown in Figure 2c) leads to an increase in the number of the hot carriers that transition to the highly excited state, and thus an increase in the number of the hot carriers participating in the hot carrier relaxation process.^[^ ^36^, ^91^ ^]^ The discontinuous increase of τ_1_ at 0.38 GPa can be attributed to the electronic state transition from ES‐I to ES‐II.^[^ ^35^ ^]^ When the pressure is above 1.58 GPa, τ_1_ disappears, indicating that the mixed states of ES‐II and ES‐III are completely transformed into ES‐III. As a result of the wide bandgap of ES‐III, the carriers cannot transition to the high excited state, so there is no hot carrier relaxation process for ES‐III. Then, τ_1_ decreases with increasing pressure from 0.98 to 1.58 GPa, which is due to the decrease in the proportion of ES‐II with hot carrier relaxation. The reduction of τ_1_ at 1.08 GPa can be attributed to the electronic state transition from ES‐II to ES‐III.^[^ ^35^ ^]^ The changing trends of τ_1_ with the pressure of CsPbBr_3_ QDs and bulk counterparts are similar.^[^ ^36^ ^]^ However, it is worth noting that CsPbBr_3_ QDs undergo an electronic state transition from ES‐I to ES‐II at 0.38 GPa,^[^ ^35^ ^]^ resulting in a discontinuous increase in τ_1_, which is not observed in the bulk counterparts.^[^ ^33^, ^35^ ^]^

In Figure 5b, τ_2_ decreases as the pressure increases from 0.16 to 0.28 GPa, which is because the density of the pressure‐induced defect states in ES‐I of CsPbBr_3_ QDs decreases, resulting in a reduction in the number of the carriers trapped by the defect states.^[^ ^91^, ^94^ ^]^ From 0.28 to 0.98 GPa, τ_2_ increases slightly, indicating that the defect state of ES‐II is less affected by pressure and its density increases little. The slight increase of τ_2_ at 0.38 GPa can be attributed to the electronic state transition from ES‐I to ES‐II.^[^ ^19^ ^]^ From 0.98 to 1.58 GPa, τ_2_ increases due to the increase in the density of pressure‐induced defect states in ES‐II, which increases the number of carriers trapped by defect states.^[^ ^51^, ^91^, ^97^ ^]^ The rapid increase of τ_2_ at 1.08 GPa is caused by the electronic state transition from ES‐II to ES‐III.^[^ ^35^ ^]^ τ_2_ disappears above 1.58 GPa suggests CsPbBr_3_ QDs are completely transformed into ES‐III, and ES‐III does not have the carrier‐trapping process of defect states. From the fitting results, τ_4_ increases slightly as the pressure increases from 1.18 to 3.38 GPa, which demonstrates that the surface state of ES‐III is less affected by pressure and its density increases little.

From 0.16 to 0.28 GPa, τ_3_ increases with the increase of pressure, as shown in Figure 5c, which is due to the decrease in the density of pressure‐induced defect states in ES‐I of CsPbBr_3_ QDs, increasing in the number of the carriers participating in radiative recombination.^[^ ^91^, ^94^ ^]^ This well explains the slight enhancement of the fluorescence at 0.28 GPa in Figure 1a. With further compression, τ_3_ decreases from 0.28 to 0.98 GPa, which is caused by the increase of the trapped carriers in the ES‐II defect state (τ_2_ increases slightly), resulting in the decrease of the carriers participating in the radiative recombination. From 0.98 to 1.58 GPa, τ_3_ continues to decrease, which is caused by the increase of trapped carrier of the ES‐II defect state (τ_2_ increases) and the increase in the number of the carriers participating in non‐radiative recombination of ES‐III (τ_5_ increases). τ_3_ disappears above 1.58 GPa, indicating that the mixed states of ES‐II and ES‐III are completely transformed into ES‐III, and there is no radiative recombination process in ES‐III. From 1.18 to 3.38 GPa, τ_5_ increases with increasing pressure, corresponding to the increase of ES‐III carriers participating in non‐radiative recombination.

**Figure 6:**
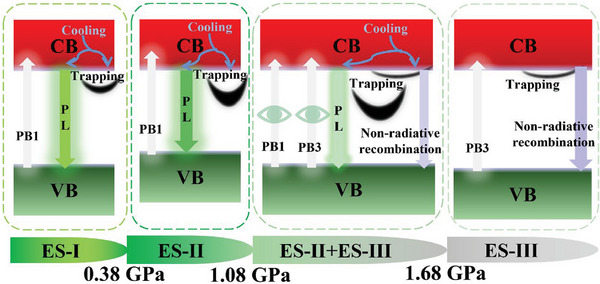
The schematic diagram for ultrafast dynamics of different electronic states in CsPbBr_3_ QDs under pressure.

Through the analysis of the pressure‐dependent carrier lifetime changes with pressure, a schematic diagram for the ultrafast dynamics of different electronic states in CsPbBr_3_ QDs under pressure is plotted, as shown in **Figure** **6**. It can be concluded that the microphysical mechanism of the pressure‐induced fluorescence enhancement in ES‐I is the inhibition of defect trapping and the promotion of radiative recombination. However, the pressure‐induced fluorescence quenching in ES‐II is due to the promotion of defect trapping and the inhibition of radiative recombination. This also validates our previous views that the reason for the pressure‐induced fluorescence disappearance of QDs is that ES‐II completely transforms into non‐radiative ES‐III under pressure, rather than the pressure‐induced amorphization in ES‐III.

## 3 Conclusion

In this study, the ultrafast dynamics in the pressure‐induced electronic state transition, fluorescence quenching, and bandgap evolution of CsPbBr_3_ QDs were explored by in situ high‐pressure steady‐state PL, Abs, and fs‐TA spectroscopy experiments. The experimental results indicate that the pressure‐induced electronic state transition path in CsPbBr_3_ QDs is different from that of the bulk counterparts. The electronic state transitions from ES‐I to ES‐II and from ES‐II to ES‐III occur in CsPbBr_3_ QDs at 0.38 and 1.08 GPa, respectively. In comparison, only electronic state transitions from ES‐II to ES‐III occur in CsPbBr_3_ bulk counterparts at 1.0 GPa. Notably, through in situ high‐pressure fs‐TA spectroscopy measurements, the ground‐state bleach of ES‐II and ES‐III can be effectively distinguished from 1.08 to 1.68 GPa, which confirms the coexistence of these two electronic states. Moreover, the pressure‐induced fluorescence quenching of CsPbBr_3_ QDs above 0.38 GPa is due to the promotion of the pressure‐induced defect state trapping process and the inhibition of the radiative recombination process in ES‐II. However, the reason for the disappearance of the pressure‐induced fluorescence is that the ES‐II with radiative recombination completely transforms into the non‐radiative recombination ES‐III, instead of the pressure‐induced amorphous in ES‐III. The fluorescence disappearance phenomenon appeared at 2.5 GPa for CsPbBr_3_ bulk counterparts, and 1.68 GPa for CsPbBr_3_ QDs. This reflects that because of the quantum confinement effect, QDs are more prone to E‐II to E‐III electronic state transition than their bulk counterparts. In addition, CsPbBr_3_ QDs have a larger bandgap tuning range (0.497 eV) from 0 to 2.08 GPa, which is 11.3 times that of the bulk counterparts. The hot carrier relaxation, defect state trapping, radiative recombination, surface state trapping, and non‐radiative recombination processes of CsPbBr_3_ QDs are all regulated by the pressure‐induced electronic state transition and bandgap evolution. The development of this study is crucial for improving the photophysical properties of perovskite materials and provides important guidance for broadening their applications in areas such as optoelectronic detectors, light‐emitting diodes, and solar cells under pressure conditions.

## 4 Experimental Section

### Sample Preparation

CsPbBr_3_ QDs (>99% purity) were purchased from Xiamen Lumansci Technology Co., Ltd. The solution concentration was 2 mg mL^−1^, and the *n*‐hexane was used as a dispersant.

### Experimental Conditions for In Situ High‐Pressure Optical Experiment

All in situ high‐pressure optical experiments were conducted at room temperature. The high‐pressure generating device used in the experiment was DAC, and the two diamond anvils were IIa ultra‐low fluorescent diamonds with an anvil surface size of 600 µm. DAC was used to prepress a piece of T301 stainless steel with an initial thickness of 250 to 150 µm for the gasket. A 500 µm diameter hole was drilled in the center of the gasket by laser as the sample chamber. Pressure calibration was performed using rubies encapsulated in the sample chamber.^[^ ^98^, ^99^ ^]^

### Experimental Conditions for Steady‐State PL Spectroscopy Measurement

The 355 nm line of a UV DPSS laser with an output power of 10 mW was used as the excitation light source. To eliminate external radiation along the emission path, interference filters and color filters were employed. For steady‐state PL spectroscopy measurements, a high‐sensitivity fiber optic spectrometer (QE Pro, Ocean Insight) with a measurement range of 355 to 1000 nm was utilized.

### Experimental Conditions for Steady‐State Abs Spectroscopy Measurement

A deuterium‐tungsten halogen lamp was utilized as the light source. For steady‐state Abs spectroscopy measurements, a high‐sensitivity fiber optic spectrometer (QE65 Pro, Ocean Insight) was employed with a measurement range of 250 to 1000 nm.

### Experimental Conditions for fs‐TA Spectroscopy Measurement

The used femtosecond laser (Newport, USA) had a laser pulse width of 100 fs, a center wavelength of 800 nm, and a repetition rate of 1 kHz. An optical parametric amplifier (OPA) generated a pump wavelength of 330 nm. The 330 nm pulses were split into two beams in a 9:1 ratio. One beam served as the pump light for sample excitation, while the other beam was directed through a computer‐controlled delay line onto an optical non‐linear transparent medium (CaF_2_) to generate supercontinuum white light. This supercontinuum white light served as the probe light to detect changes in absorbance induced by the pump light in the sample. The probe and pump lights were kept overlapped in the sample chamber.^[^ ^57^, ^58^, ^77^ ^]^ Detection was carried out using a charge‐coupled device (CCD) detector. The acquired fs‐TA spectroscopy was globally fitted using the CarpetView software.

## Conflict of Interest

The authors declare no conflict of interest.

## Supporting information

Supporting Information

## Data Availability

The data that support the findings of this study are available from the corresponding author upon reasonable request.
