# Supplementary material for: Ultrafast Dynamics Across Pressure‐Induced Electronic State Transitions, Fluorescence Quenching, and Bandgap Evolution in CsPbBr3 Quantum Dots
Source: Adv Sci (Weinh). 2024 Feb 2;11(14):2308016. doi: 10.1002/advs.202308016 (PMC11005694; doi:10.1002/advs.202308016)
Supplement: Supplementary file 1 — Supporting Information [file ADVS-11-2308016-s001.pdf]

ADVANCED SCIENCE
Open Access

Supporting Information

for *Adv. Sci.*, DOI 10.1002/advs.202308016

Ultrafast Dynamics Across Pressure-Induced Electronic State Transitions, Fluorescence Quenching, and Bandgap Evolution in $CsPbBr_3$ Quantum Dots

Lin Chen, Ya Chu, Xiaxia Qin, Zhijian Gao, Guozhao Zhang, Haiwa Zhang, Qinglin Wang, Qian Li, Haizhong Guo*, Yinwei Li and Cailong Liu**

Supporting Information

Ultrafast Dynamics across Pressure-Induced Electronic State Transitions, Fluorescence Quenching, and Bandgap Evolution in $CsPbBr_3$ Quantum Dots

Lin Chen[#], Ya Chu[#], Xiaxia Qin, Zhijian Gao, Guozhao Zhang, Haiwa Zhang[*], Qinglin Wang, Qian Li, Haizhong Guo[*], Yinwei Li, and Cailong Liu[*]

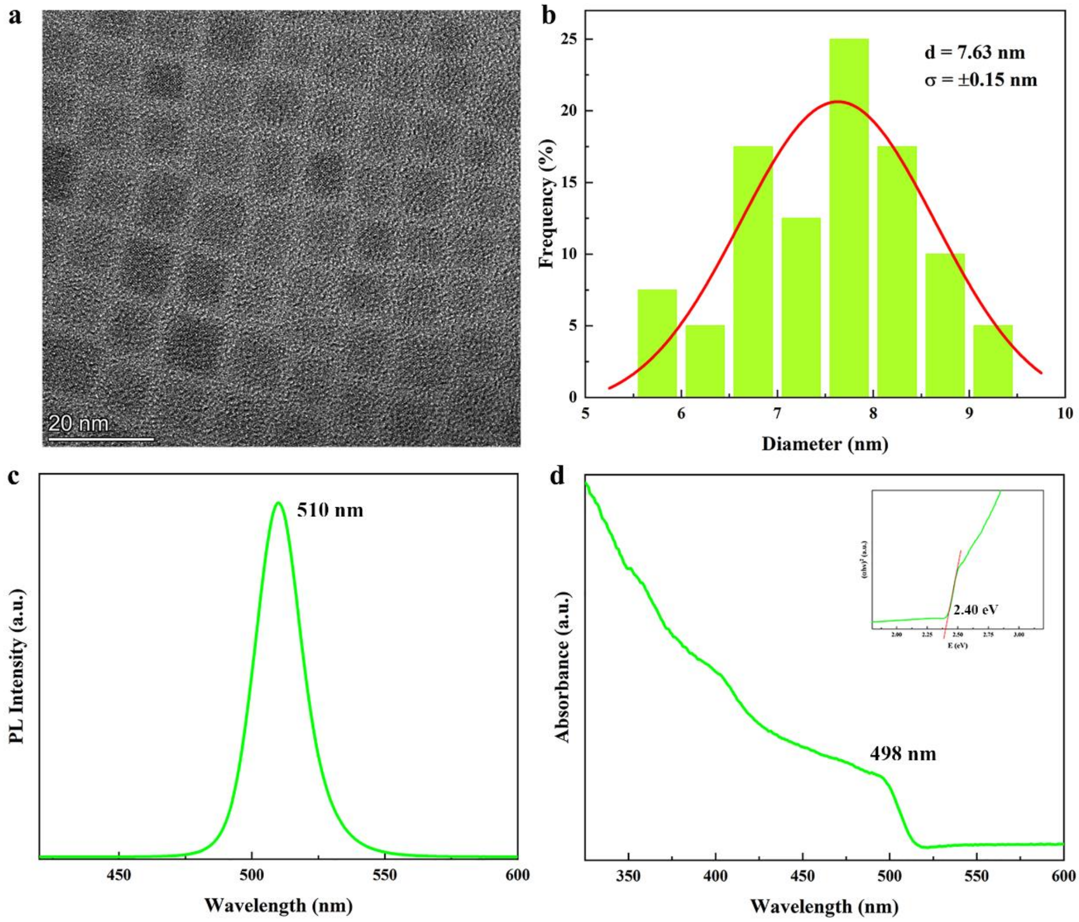

Figure S1. Characterization of $CsPbBr_3$ QDs at ambient pressure. a) High-resolution transmission electron microscopy image. b) Corresponding size distribution map obtained by Gaussian fitting. c) Steady-state PL spectrum. d) Steady-state Abs spectrum and the inset show the calculated bandgap Tauc plot at ambient pressure.
